# Disparities in End-of-Life Outcomes: A Demographic and Geographic Analysis of Endometrial Cancer Deaths in the United States

**DOI:** 10.7759/cureus.81986

**Published:** 2025-04-10

**Authors:** Sai Shreya Surapaneni, Gaurang N Narayan, Swati G Kapsikar, Rupesh J Gawadkar, Sana U Rajkotwala, Akash Ingle, Saniah M Kazi, Akanksha A Thakre, Megha R Awale, Monica Saini

**Affiliations:** 1 Obstetrics and Gynaecology, Kamineni Institute of Medical Sciences, Narketpalle, IND; 2 Obstetrics and Gynaecology, Squad Medicine and Research - OBGY Wing, Tiruchirappalli, IND; 3 Obstetrics and Gynaecology, Indira Gandhi Government Medical College and Hospital, Nagpur, IND

**Keywords:** death, demographic factors, end-of-life care, endometrial neoplasms, healthcare disparities, hospice care

## Abstract

Introduction: Endometrial cancer poses a significant public health challenge globally. With an increasing incidence, understanding end-of-life outcomes becomes crucial in navigating this landscape. This study aims to explore disparities in endometrial cancer death locations across demographic and geographic parameters.

Methodology: Using the Centre for Disease Control and Prevention - Wide-Ranging Online Data for Epidemiologic Research (CDC WONDER) database, data on endometrial cancer deaths were retrieved and analysed. Logistic and linear regression models were employed to identify predictors and trends in place of death.

Results: The study identified 90,140 endometrial cancer deaths from 1999 to 2020. Age-specific analysis revealed higher mortality rates in the 64-75 years age group. Geographically, the South reported the highest mortality rates. White individuals exhibited higher death rates across all settings. Age, race, and geographic disparities were evident in endometrial cancer death locations.

Conclusions: Understanding end-of-life outcomes in endometrial cancer is crucial for improving patient care. Tailored interventions addressing demographic and geographic disparities are essential for ensuring equitable and dignified end-of-life care for individuals with endometrial cancer.

## Introduction

The natural conclusion of life, whether expected or unexpected, is an integral aspect of human existence. Understanding the variables that can have a major impact on a patient's end-of-life journey is crucial as we work to guarantee that patients have a dignified trip [1-4]. The location of death - at home, in a hospice, nursing home, hospital, or other medical facility - is a crucial consideration. High-quality end-of-life care, including symptom control through palliative care, is frequently offered in hospitals and assisted living institutions [5-7]. However, the level of care may not always be consistent in home or hospice settings, potentially resulting in distressing experiences for patients. Despite advancements in healthcare, disparities persist in where individuals ultimately pass away. Further, this disparity cannot be generalised for all the disease entities [6-8]. One such disease entities that lack scientific literature on the evaluation of such disparities is malignant neoplasms of the endometrium.

Endometrial cancer, recognized as the fifth most prevalent cancer among women globally, manifests as a significant public health concern. In 2012, a staggering 320,000 new cases were diagnosed, constituting 4.8% of all cancers in women [9]. Within the spectrum of gynaecologic cancers, endometrial cancer stands out as the most common, with an annual incidence surpassing 380,000 [10]. Despite its prevalence, a notable aspect of hope exists - nearly 70% of patients receive an early-stage diagnosis, contributing to the overall positive prognosis associated with this malignancy [11]. Furthermore, there has been a consistent decrease in the risk of death from endometrial cancer over the past four decades [12].

Navigating the landscape of endometrial cancer involves a pivotal reliance on surgical interventions, encompassing procedures such as bilateral salpingo-oophorectomy, total hysterectomy, and lymph node assessments. The intricacies of post-surgical care, including the decision-making process for adjuvant therapy, introduce a nuanced dimension that considers factors such as histology and stage [13]. Amidst this complex framework of diagnosis and treatment, this research aims to broaden the perspective by delving into the end-of-life outcomes of individuals grappling with endometrial cancer. This study seeks to unravel the intricacies of these disparities across age, race, and geographic regions, contributing to a nuanced comprehension of the multifaceted nature of endometrial cancer outcomes. By doing so, we aim to inform and enhance end-of-life patient support, fostering more personalized, dignified, and compassionate care for individuals affected by this formidable disease.

## Materials and methods

Conducted in December 2023, this web-based observational study employed a facility-based cross-sectional design over a four-week period. Data were sourced from the Centre for Disease Control and Prevention - Wide-Ranging Online Data for Epidemiologic Research (CDC WONDER) database, which provides comprehensive information on the underlying causes of disease-related deaths. Accessible and publicly available, this database facilitated the retrieval of details on death certification, place of death, and demographic characteristics of patients in the USA. The data spans from 1999 to 2020 and is easily accessible online [14].

All the data were acquired on a single day, focusing on individuals of diverse races who succumbed to endometrial malignant neoplasms from 1999 to 2020. The database query specified “1999-2020: Underlying Cause of Death by Bridged-Race Categories”, targeting the specific International Classification of Diseases, Tenth Revision (ICD-10) code for endometrial malignant neoplasms (C54.1). The variables examined encompassed broad patient-related characteristics, such as year of death, place of death, age, race, and US census region, which were subsequently organized and stratified. Age was segmented into 10-year intervals, including individuals of all races. Geographic locations were categorized according to the US census regions: Northeast, Midwest, South, and West. The data were additionally arranged according to the location of death. For the purpose of this study, all recorded places of death were classified into three groups: home or hospice (including the descendant’s home and hospice facility), medical facility or nursing (covering inpatient, outpatient, emergency room, dead on arrival, status unknown, nursing home/long-term care), and others (encompassing other designations and cases where the place of death was unknown).

The information was transferred to a Microsoft Excel Sheet (Microsoft Corporation, Redmond, WA) and subjected to statistical analysis through R programming software (R Core Team, Vienna, Austria).

The summarized data encompassed total deaths across all years and races, with both aggregated and individual data expressed as frequencies. To assess disparities, a univariate logistic regression model was employed, which allowed for the examination of the relationship between the binary outcome (death vs. no death) and predictor variables, such as year and race. Odds ratios (ORs) were calculated to quantify the strength of these associations, providing insight into how the odds of death varied across different racial groups and over time.

Additionally, a linear regression model was applied to assess the trend in deaths over time. This model facilitated the calculation of R² values, which indicate the proportion of variance in the total deaths that could be explained by the year of observation. The regression model was used to generate trend lines, which were then utilized to project future death rates. These projections provided estimated death counts for the next five years based on the historical data, helping forecast potential disparities and shifts in trends. To ensure robustness, model assumptions such as linearity, independence, and homoscedasticity were checked. Confidence intervals for the ORs and R² values were also calculated to provide an estimate of the precision of the results. This approach not only quantified past trends but also allowed for predictive insights into future disparities in death rates.

## Results

The aggregate data encompassing the total number of individuals who succumbed to malignant neoplasms of the endometrium in the USA from 1999 to 2020, as extracted from the CDC WONDER database, amounted to 90,140. Table 1 categorizes these deaths based on the place of occurrence - home/hospice care, medical care/nursing facility, and others. The majority of patients (n=28583, 31.70%) fell within the 65-74 age group, dominating across all designated places of death. For malignant neoplasms of the endometrium that are oestrogen-dependent, the risk of death follows a "Bath-tub" or "U-shaped" distribution, increasing with age, peaking and then declining. Geographically and racially, the South region (n=30532, 33.87%) and the White race (n=72226, 80.13%) exhibited higher reported deaths from malignant endometrial neoplasms.

**Table 1:** Basic sociodemographic details and categories of deaths based on the place of occurrence - home/hospice care, medical care/nursing facility, and others

Variables	Home or Hospice	Medical Facility or Nursing	Others
Ten-Year Age Groups	(n=44765)	(n=39890)	(n=5432)
25-34 years	110	170	0
35-44 years	682	759	75
45-54 years	2761	2815	333
55-64 years	9679	8666	1033
65-74 years	14869	12079	1635
75-84 years	11437	9749	1541
85+ years	5227	5652	815
Census Region	(n=44,774)	(n=39,925)	(n=5441)
Census Region 1: Northeast	8630	10,522	768
Census Region 2: Midwest	10,395	10,721	1365
Census Region 3: South	16,183	12,038	2311
Census Region 4: West	9566	6644	997
Race	(n=44774)	(n=39913)	(n=5446)
American Indian or Alaska Native	159	187	16
Asian or Pacific Islander	1285	1104	136
Black or African American	6789	7397	834
White	36,541	31,225	4460

Table 2 outlines the results of the univariate logistic regression, identifying various predictors for deaths at home/hospice in patients with malignant endometrial neoplasms. Using the age group of >85 years as reference, patients aged 55-64 years, 65-74 years, and 75-84 years had a higher likelihood of experiencing such deaths. When examining geographic locations with patients from the Northeast region as the reference, individuals from all other regions were more inclined to die at a home/hospice setting. Additionally, when compared to American Indians or Alaska Natives, those from the Pacific islander/Asian race and the White race had a higher likelihood of dying in a home/hospice-based setting, with the Black/African American race as the reference.

**Table 2:** Univariate logistic regression, identifying various predictors for deaths at home/hospice in patients with malignant endometrial neoplasms The significant p-values (<0.05) are marked with an asterik (*).

Variables	Univariate Logistic Regression			
	Odds Ratio	95% Confidence Interval	P-value	
Age				
25-34 years	0.801	(0.628, 1.02)	0.072	
35-44 years	1.012	(0.909, 1.127)	0.832	
45-54 years	1.085	(1.019, 1.155)	0.011*	
55-64 years	1.235	(1.179, 1.293)	<0.001*	
65-74 years	1.341	(1.285, 1.401)	<0.001*	
75-84 years	1.253	(1.198, 1.311)	<0.001*	
85+ years	1.0 (Reference)			
Census Region				
Census Region 1: Northeast	1.000 (Reference)			
Census Region 2: Midwest	1.125	(1.083, 1.169)	<0.001*	
Census Region 3: South	1.475	(1.423, 1.529)	<0.001*	
Census Region 4: West	1.638	(1.572, 1.707)	<0.001*	
Race				
Asian or Pacific Islander	1.256	(1.155, 1.367)	<0.001*	
American Indian or Alaska Native	0.95	(0.77, 1.172)	0.63	
White	1.241	(1.198, 1.286)	<0.001*	
Black or African American	1.000 (Reference)			

**Figure 1:**
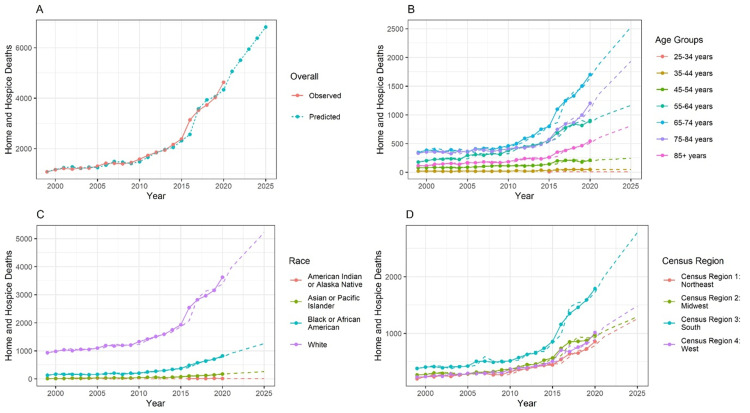
Autoregressive integrated moving average (ARIMA) model of forecasted data for the upcoming five years The forecasting is done from 1999 to 2025. The training data are available from 1999 to 2020. Thus, the prediction is done for another five years. In the line chart, the lines represents the observed data. The dotted line represents the forecasted data. The method used is the ARIMA model.

Figure 1A depicts the total number of home/hospice deaths from 1999 to 2020, revealing a consistent year-by-year increase. Although the number of deaths remained relatively constant between 2000 and 2010, a noticeable upward trend emerged afterwards. It must also be noted that an observable upward trend is predicted in the future. In Figure 1B, an age-group specific analysis of home/hospice deaths is presented, highlighting that the age group 65-74 years recorded the highest number of deaths. Figure 1C illustrates the distribution of deaths among various races, with the White race documenting the highest number and American Indian/Alaskan Natives the lowest. In Figure 1D, deaths across different census regions are examined, revealing the South as having the highest number.

## Discussion

The mortality trends observed in endometrial cancer underscore the complex interplay between various risk factors, demographic characteristics, and healthcare disparities. This analysis revealed several noteworthy findings that shed light on the epidemiology and prognosis of endometrial cancer. Firstly, this age-specific analysis identified a notable concentration of deaths in the 64-75-year age group across all settings, including medical or nursing facility, home or hospice, and other categories. Conversely, the 24-34-year age group exhibited the lowest mortality rates in these settings. Importantly, among all age groups, the study observed a higher frequency of fatal outcomes in women over 80 years old with endometrial cancer and high BMI, aligning with findings by Liu et al. [15]. This emphasises the significance of considering age and BMI as significant factors in endometrial cancer outcomes, warranting targeted interventions and personalised care strategies for elderly women with elevated BMI to improve survival outcomes.

Examining mortality trends based on census regions revealed a striking pattern, with the highest deaths consistently observed in census region 3 (South) across all categories. In contrast, census region 1 (Northeast) exhibited the lowest mortality rates for home or hospice and other settings. These regional disparities underscore the importance of considering geographic variations in healthcare access, quality of care, and socioeconomic factors that may contribute to divergent outcomes.

Furthermore, this study corroborates existing literature on racial disparities in endometrial cancer outcomes, with the highest deaths occurring in white women across all settings. This finding is supported by the research conducted by Huang et al., which highlights the challenges faced by black women in receiving evidence-based care and the persistence of racial disparities in outcomes, even with improved care quality [16].

The findings of Huang et al. underscore the critical role of evidence-based care in mitigating, though not eliminating, racial disparities in endometrial cancer outcomes. Understanding these disparities and their relationship to the quality of care is crucial for developing targeted interventions aimed at improving survival rates among black women.

Finally, the rising incidence of endometrial cancer among young adult women is a concerning trend that warrants further investigation. The concurrent increase in obesity prevalence among young girls and women suggests a potential link between obesity and early-onset endometrial cancer. These findings underscore the importance of early detection and screening efforts, particularly among high-risk populations presenting with abnormal bleeding symptoms [17-19].

Limitations

The utilization of data from CDC WONDER, while constituting a valuable resource for this study, introduces inherent limitations. The integrity and precision of the dataset hinge upon prevailing reporting practices, coding procedures, and data collection protocols. The inherent variability in these elements introduces the potential for biases or inaccuracies that may exert influence on the robustness of these findings.

A noteworthy constraint within this investigation lies in the unavailability of the most recent data spanning from 2021 to 2023. The reliance on information up to the year 2020 imposes a constraint on our capacity to encompass the latest developments or alterations in the dynamic landscape of mortality trends in endometrial cancer. Furthermore, an additional constraint emerges from the lack of sub-categorization for endometrial cancer within this study. The absence of differentiation based on specific subcategories represents a potential oversight, neglecting nuances that could impart a substantial impact on mortality trends.

While the study underscores age-related patterns and the correlation between elevated BMI and mortality, it falls short of delving into specific considerations associated with other demographic or clinical factors. Elements such as comorbidities, treatment modalities, and socioeconomic status, which wield potential influence on mortality, have not been comprehensively addressed within the scope of this analysis. Moreover, the study, in recognizing racial disparities in endometrial cancer outcomes and their linkage to quality of care, refrains from an exhaustive exploration of the intricate mechanisms underpinning these disparities. A comprehensive understanding of the intricate interplay involving socioeconomic, cultural, and healthcare system factors contributing to these disparities necessitates a dedicated investigation that extends beyond the confines of this current study.

## Conclusions

This study highlights significant trends in end-of-life outcomes for endometrial cancer across demographic groups and care settings. Mortality was highest among women aged 64-75 years, with the lowest rates observed in those aged 24-34 years. Additionally, women over 80 years with high BMI exhibited increased fatality rates, reinforcing the need for personalized, weight-sensitive care strategies for this high-risk group.

Geographic disparities in mortality were evident, with census region 3 (South) consistently reporting the highest deaths and region 1 (Northeast) the lowest. These findings underscore the urgent need to address regional inequities in healthcare access, quality, and socioeconomic determinants. Future efforts should prioritize targeted interventions for elderly, high-BMI patients, bridge geographic and racial disparities, and integrate multidisciplinary and palliative care approaches to ensure equitable, patient-centered end-of-life care.
